# It’s Sunny, Be Healthy? An International Comparison of the Influence of Sun Exposure and Latitude Lines on Self-Rated Health

**DOI:** 10.3390/ijerph18084101

**Published:** 2021-04-13

**Authors:** Sandra Jaworeck, Peter Kriwy

**Affiliations:** Institute for Sociology, Chemnitz University of Technology, 09107 Chemnitz, Germany; peter.kriwy@hsw.tu-chemnitz.de

**Keywords:** sun, latitude lines, self-rated health, vitamin D, international comparison

## Abstract

The positive impact of sunshine on self-rated health is well known. For the first time, the relationship between sunshine and self-rated health is examined in the context of latitude lines in international comparison. The further people live from the equator, the lower sun exposure (UVB exposure) and the more often they experience a vitamin D deficiency. UVB exposure decreases with degrees of latitudinal lines, and in addition to that, sunshine duration is shorter in northern countries. In order to consider the connection, sunshine duration and degree of latitude lines were manually enriched from the German Meteorological Service (Deutscher Wetterdienst) to the International Social Survey Programs (2011): Health and Health Care and analyzed with a logistic multilevel model, as well as the inclusion of sunshine duration as a mediator. If sunshine hours, as well as latitude lines, are considered separately in models, both show a statistically significant effect. Together in one model, the sunshine hours lose their relationship and additionally there is no mediation. This suggests that the location of the region is the decisive component when considering self-rated health. Furthermore, an interaction between age and sunshine hours as well as latitude lines is also shown.

## 1. Introduction

The positive effect of sunshine on mood and well-being for human beings seems to be common knowledge. In the short term, one reason could be that good weather increases motivation in people [1]. In the long term, a lack of vitamin D can have a negative impact on health, for example by reducing muscle development or by weakening the immune and cardiovascular system [2,3].

Preserving one’s health has become a moral imperative [4] and reportedly health is one of the most important values for people [5,6]. Apart from this, the health and well-being of humans is one of the 17 central sustainability goals of the United Nations [7]. According to the definition of the World Health Organization, health is “a state of complete physical, mental and social well-being and not merely the absence of disease or infirmity” [8]. Following this definition, an analysis of the influencing factors of health has to include both physical and mental aspects.

### 1.1. Sunlight and Sun Exposure

Today it is known that vitamin D, the so-called sun hormone is needed by almost all organs and cells of the body [9]. Increased exposure to the sun should therefore be associated with improved health benefits. For perceptive reasons, which relate to the fact that people enjoy sunlight when they see it and for reasons which relate to the health-benefiting effects of UVB (ultraviolet B, electromagnetic radiation with wavelengths between 290 and 320 nanometers).

It is beyond doubt that there are many factors that affect health, including gender, age, education, but also the frequency of locomotion and exercise, dietary behavior, and substance use of alcohol, cigarettes, and illegal drugs [10,11]. However, people living in regions above latitudes 35° N and below 35° S are unable to produce enough vitamin D, regardless of biological precondition, behavior or annual sum of sunshine hours [12] and this has been rarely acknowledged within the literature. Lucas and Lawless for example investigate the connection between weather conditions and life satisfaction. Their analysis integrates various potential influence factors, including temperature, rain, cloud density, and air pressure yet does not account for sunshine duration [13]. The authors merely make a reference to Howarth and Hoffman, who investigate the effect of sunshine duration on a rather small sample of 24 men [14]. Abeliansky and Strulik explore the connection between the time of birth, aging, and health. They operationalize sun exposure with the rough indicator of seasons [15].

The present paper tries to separate sunlight as a source of well-being from the effect that sun exposure (UVB) as represented by latitude lines has on the vitamin D stock. Therefore, the following research questions are examined:

*Do people rate their health worse if they live further away from the equator? Does sunshine have a mediating effect in this respect?*

### 1.2. Self-Rated Health

When people assess their health (self-rated health), they evaluate whether they are currently experiencing any health problems regarding mood and well-being [16]. In doing so, they apply three different kinds of knowledge: schematic knowledge, episodic knowledge, and information about changes. Schematic knowledge is information based on previous diagnoses with an assessment of how healthy one will be in this context. Self-assessment of one’s own health-related behavior such as physical exercise and nutrition also serves as a guiding tool [17]. Episodic knowledge refers to lightsome times of good health and memorable events such as a hospital stay [17,18]. Information about changes relates to people comparing their current health state to that of a younger self. They might for example experience healing processes that take longer than in the past, or that the symptoms of an existing illness have subsided [18]. These aspects, or a selection of them, are evaluated simultaneously in order to reach an assessment of one’s own subjective health [17,18].

Self-rated health is a powerful indicator for various fields and as a measure of health equally suited as objective measurements [19]. Self-rated health reflects the expected risk of death better than measurements of objective diseases [17]. A meta-study shows that in eight out of nine cases, a question on subjective health predicts mortality as good as an objective measure [20]. Roughly 50% of the variance in self-rated health can be explained by functioning, diseases, pain, mental health, and behavior, with functioning and diseases being the most important aspects [21].

### 1.3. Sunlight as a Source of Mental Influence

The sun is the most available natural light source. When the sun shines, many people claim to be in a good mood [1] or less tired [22]. Artificial in addition to natural light sources could influence context or initial situation (mood as well as well-being) and thus have an effect on health, which leads us to the first hypothesis:

**H1.**  *The more sunshine in a region the better the health.*

### 1.4. Regions and Their Location (Latitude Line) as a Proxy for Vitamin D

Vitamin D is metabolized from cholesterol through the skin and liver under influence of sun exposure, more precisely the UVB component [9]. Whereas it was assumed for a long time that the main role of vitamin D is to maintain calcium homeostasis and bone health [23] it is known today that the sun hormone is needed for and influences almost all organs and cells of the body [9]. In place of sun exposure, the body can produce vitamin D through the consumption of fatty fish, eggs, and dairy products. For a substitute of a sufficient amount of sunshine, a person needs very large quantities of it, for instance, two large portions of salmon or 20 eggs a day. Especially in more northern countries of Europe, a lot of vitamin D-rich food is eaten, which can lead to possible compensation. Chemically produced food supplements can also provide an alternative [24,25]. If individuals cannot take advantage of one of these options, sun exposure is the main source of vitamin D [26].

Insufficient sun exposure of the skin over a long period of time results in a state known as vitamin D deficiency [2,3]. Bazzano et al. assume that people above 35° N and below 35° S latitude line have vitamin D deficiency due to insufficient sun exposure [12]. The exact amount of sunlight depends on the latitude and height of the location as well as the time of year (if seasons are given for the region). The earth’s axis is inclined against its orbital plane around the sun by the angle ε = 23.5°. The angular distance of the sun from the equatorial plane changes during a year between +23.5° on June 21 and −23.5° on December 22. Due to these conditions, sun exposure of varying intensity is to be expected throughout the year. This is strongest at the equator because the angle there is constantly the smallest. The larger the angle and thus the further a country is from the equator and the lower the sun exposure. More can be read about the formation of vitamin D through sun exposure in Barger-Lux and Heaney [27]. We test the following hypothesis:

**H2.**  *Regions further away from the equator are associated with lower prospects for good population health.*

### 1.5. The Connection between Sunshine and the Location (Latitude Lines)

Sun exposure decreases with degrees of latitude and sunshine duration is lower in northern countries than in countries that are close to the equator as well as in winter months due to fewer hours of sunlight. The central question here is whether longer hours of sunshine duration can mediate the influence of reduced UVB exposure as represented through latitude lines on health-related opportunities, which leads us to the acceptance of mediation:

**H3.**  *Sunshine duration mediates the effect of latitude lines on population health.*

The greater the distance to the equator, the less UVB exposure can be expected [28] no matter whether deviations are considered upwards or downwards. Reduced health prospects are expected in both cases. In terms of the sequence of influences over time, the number of sunshine hours can be located between latitude lines and health prospects.

Although no longitudinal design is available, it is possible to test for mediation here, because the latitudinal lines are permanent and do not vary. Thus, only sunshine duration can modify the influence of the latitudinal line on health-related opportunities.

Hypothesis 3 is significant because it can assess the relationship between latitudinal lines and sunshine hours in a dependent model. In light of it, it becomes clear whether the sunshine hours or the location (latitude line) have effects on health. Therefore, this hypothesis combines the first two and can subsequently show the true effect.

### 1.6. Control Variables

The selection of control variables is based on theoretical assumptions about their connection to self-rated health and previous empirical findings. At the individual level, the control variables are confidence in one’s own health care system, smoking behavior, sports behavior, working hours, education, age, and sex. With regard to possible country differences, domestic general government health expenditure (% of GDP) and skin color are used to monitor the contextual level. Furthermore, the season is controlled.

There is a significant and positive relationship between institutional trust in the health system and self-rated health [29]. Therefore, trust in one’s own health system is integrated.

The negative consequences of tobacco consumption for physical health have been confirmed many times [30,31]. Smoking affects physical health not only directly, but also indirectly by influencing health-sustaining factors such as diet, sport, and drinking behavior. Smokers eat healthy food less often, exercise less, and have a higher prevalence of alcohol dependence and risk consumption than non-smokers and former smokers [32]. Furthermore, harmful behaviors build up with an increase in severity of nicotine dependence [32,33,34,35,36,37,38,39]. In addition to all that, smoking cigarettes disrupts vitamin D metabolism in the lungs [40].

Physical activity not only helps in the treatment and rehabilitation of various diseases but can also be used preventively. People who are physically active face fewer health problems and consider themselves to be healthier when compared to those who do not engage in physical activity [41].

The working hours are recorded to control the individual sun exposure as well as possible. The assumption behind this is that people who work a lot are less likely to be exposed to sunlight. In general, employment is positively correlated with health. The day is structured through employment, the experience of meaningful activity increases positive social feedback, and sometimes physical activity is encouraged. Together, these aspects are positively associated with health.

Educational background is positively associated with health prospects [42] and a positive correlation between education and mental health has been corroborated as the share of people with mental impairments decreases among those with higher educational status [43].

Longitudinal studies have shown that self-rated health changes with increasing age [44]. Whereas physical health objectively deteriorates with age, people tend to consider themselves to be healthier than they are. In addition to an increased risk of morbidity due to biological aging processes, changes in lifestyle, and health-related behavior can also explain this finding [45]. As people age, the importance of good health increases because health turns into a more fragile asset due to the development of chronic illness and physical complaints [44]. Age is also a major risk factor in vitamin D deficiency due to decreased sunlight exposure, dietary intake, and skin thickness. Therefore, interactions between sunshine hours and age (H1 and H3), as well as latitude lines (H2 and H3), are included.

Men and women differ in terms of mortality, morbidity, health behavior, but also in their self-rated perception of health and illness [46]. On the basis of survey results, the gender health paradox must also be considered, which describes the observation that men consider themselves to be healthier than women although the latter have a longer life expectancy [47,48].

Since there is currently no cross-country classification of the health care system, domestic general government health care expenditures (as a % of gross domestic product) are used here in order to be able to control for country differences. The advantage is that, on the one hand, private health expenditures are not considered and, on the other hand, a relation to the gross domestic product of the respective countries is given.

Furthermore, we control for skin color in the different countries. Skin color is associated with perceived discrimination, stress, and socioeconomic status. These in turn are related to one or more health outcomes [49]. There is also a connection between skin color and vitamin D absorption.

Finally, we control for the season in which the survey took place. Temperature and precipitation were to be additionally controlled, but no correlations could be found, which is why they were taken out again.

## 2. Materials and Methods

### 2.1. Materials

The dataset which we base our analysis on is the International Social Survey Programme (ISSP): Health and Health Care—ISSP 2011, founded in 1984 and currently consisting of 42 member states worldwide [50]. The survey takes place annually and has changing focus topics. So far, 2011 is the only survey wave with a focus on health and health care. Between February 2011 and April 2013, a total of 55,081 people from 32 countries were interviewed. In addition, the ISSP—Health and Health Care was complemented with latitude lines and climate data of the monthly sunshine duration from the place of residence and the time of the survey from the German Meteorological Service [51]. These are validated data that are subject to routine quality control [52]. The additional process-generated data allow sun exposure and health to be analyzed in an international comparison.

The data extension by monthly sunshine duration was carried out according to the following criteria: If possible, the three largest regions (measured by the number of respondents in the region) of a country should be considered or at least ten data points per country should be available. Simultaneously, small-scale information on local and synchronous sun exposure is required. If a region had to be excluded because no information on sunshine duration was available, we integrated data on the nearest larger region. Four countries had to be excluded for differing reasons. First, there was no question on self-rated health in the Taiwan survey instrument. Secondly, no information on the months of data collection was available for Bulgaria. In the third case, it was not possible to access validated climate data of the DWD for Norway. Lastly, the Belgium case had to be excluded due to the small number of cases as only data on one weather station with 26 participants in Brussels was available. The net sample consists of 28 countries with a total of 18,006 people interviewed. The region with the lowest number of cases has 155 respondents and the largest has data from 2195 persons, the average number of cases per region is 643 individuals. Possible systematic peculiarities due to the reduction of cases are examined as well. For example, it could be assumed that more weather stations are available in more prosperous regions. The sub-sample of 18,006 people does not differ in self-rated health status, working hours, smoking and sports behavior, age, and sex from the total number of 55,081 cases.

### 2.2. Operationalization

The dependent variable of the analysis is self-rated health. The wording of the question is: In general, would you say your health is…; response categories: excellent, very good, good, fair, poor (or can’t choose). Because of the skewed distribution of the variable and the widely varying frequencies of expression in the categories, we dichotomized the variable to obtain the best estimations. The five-digit scale is dichotomized by using the evaluation strategy of the meta-analysis from Kondo et al. [53], which allows one to compare people who assess their health as good to excellent with people who place themselves in the categories from fair to poor (hereinafter: not good).

The monthly sunshine duration is derived from the Climate Data Center of the German Meteorological Service [51] and then divided by 100. Accordingly, the interpretation is based on a scale of 100 h of sunshine per month.

Sufficient vitamin D intake occurs only between the 35° N and 35° S latitude lines [12]. Therefore, these countries were grouped together and compared with countries further away from the equator.

Confidence in the health care system is measured with the following question: In general, how much confidence do you have in the health care system in (country)?; response categories: complete confidence, a great deal of confidence, some confidence, very little confidence, no confidence at all (or can’t choose). The five-digit scale is dichotomized into the categories high (complete and great deal) and low (some and below).

Smoking behavior is monitored by the question: Do you smoke cigarettes, and if so about how many cigarettes a day? and is recoded in four categories differentiating between persons who have never smoked, former smokers, smokers with up to ten cigarettes a day, and smokers with more than 10 cigarettes a day.

Sports behavior is surveyed with the question: How often do you exercise physically for at least 20 min so that you sweat or breathe more than usual? and divided into the categories never, rarely (once or several times a month) and frequently (several times a week and daily).

The number of hours worked was open-ended and is included in the analysis as a metric variable. It should be noted that all persons who do not work are also included in the analysis using the same variable with a value of zero.

To measure education, the aggregated form of the International Standard Classification of Education (ISECD) is used, which has become a well-established tool for national and international research and summarizes educational levels in the educational groups low, medium, and high [54].

Age was queried openly and limited in the analysis to persons between 18 and 82 years of age in order to define a uniform upper and lower limit (18 years of the actual possible characteristics) to reduce possible survival effects (distortions in favor of survivors) [55]. We wanted to include an additional survivorship variable, but it resulted in too high multicollinearity and therefore had to be removed again.

Sex was asked dichotomously by asking directly for it (woman or man). No alternative information could be given. Only persons who indicated their gender were included in the analysis since a rather negligible number of 11 persons did not want to assign themselves.

Domestic general government health expenditure (as % of gross domestic product) data are enriched using the word bank group [56]. These are expressed as a percentage, with the lowest value coming from the Philippines at 1.11% and the highest from Japan at 8.89% and are included as a metric variable.

Skin color is classified according to the procedure of Parra et al., see Figure 1 in [57]. In total there were eight levels from one white to eight black. Due to too high multicollinearity in the attempt to include the variable as categorical, we fall back quasi-metric in order to avoid giving up control of this important variable.

Lastly, we looked at when the surveys were conducted (in which month) and then adjusted the seasons (spring, summer, fall, and winter) according to the southern and northern hemispheres. Furthermore, there is a category no seasons for regions of countries that are too close to the equator to have any seasons.

**Figure 1:**
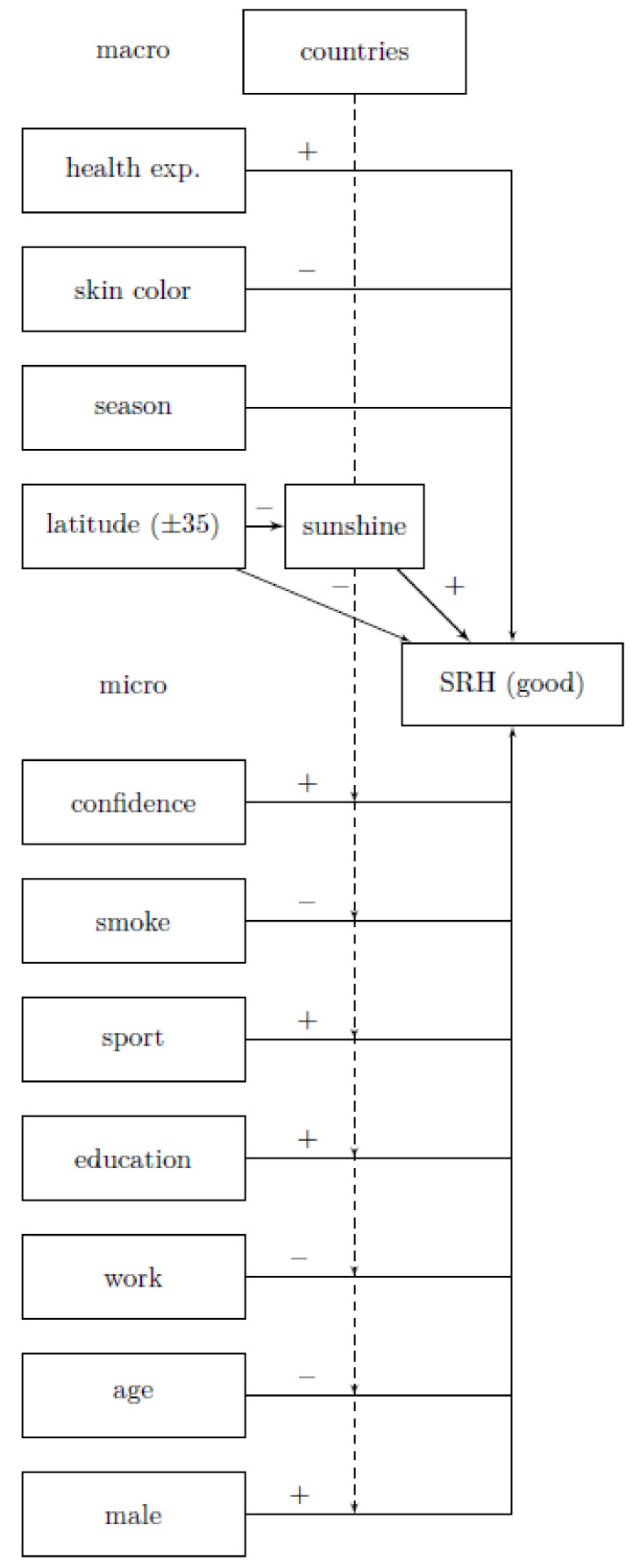
Description of relevant mechanisms related to sun exposure and self-rated health, health exp. = domestic general government health care expenditures (as a % of gross domestic product), interaction from age and sunshine hours not shown, own representation, [±35] means all values above 35° N and below 35° S parallel.

Each person on the micro-level can be assigned to a country (macro-level, see Figure 1). On this level, in addition to monthly sunshine duration, latitudes and the Human Development Index are also considered. The country-specific context furthermore affects individual characteristics, as indicated by dotted arrows. The variables needed for the hypothesis tests are located at the macro level. The variable *sunshine* represents the mediator, while a direct arrow from latitude via *sunshine* leads to self-rated health (SRH). Therefore, while we have no information on how much a single person was exposed to the sun, we assume that the number of people who benefit from sun exposure increases with increasing sunshine duration in a country.

### 2.3. Methods

**Figure 2:**
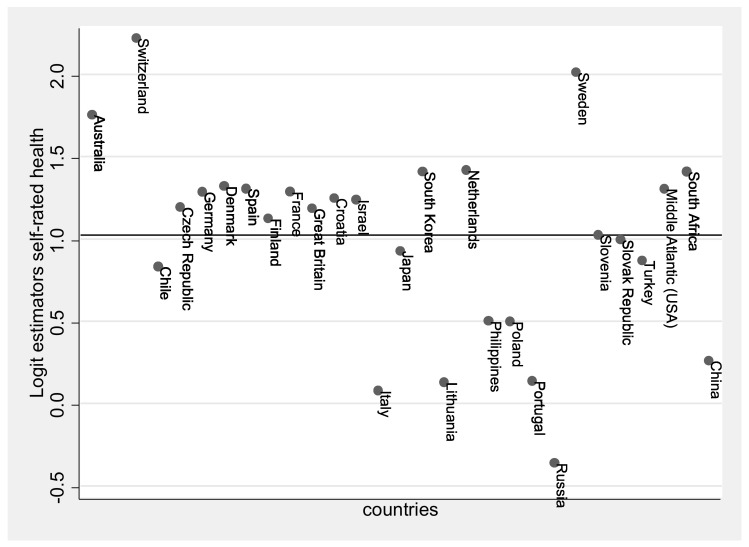
Variance of countries in self-rated health represented with Logit estimators, estimate across all countries shown as a line (Logit estimation for all = 1.025), own calculation and representation.

The comparison of latitude lines under the mediation of sunshine duration and their effect on self-rated health is analyzed by comparing countries and their differing monthly sunshine duration. Integrating country variables into the analysis is important because it is likely that country affiliation (health care system, politics, etc.) has an effect on individual persons. A multilevel model is used to perform a country-weighted analysis. Weighting is applied in order to control for group size differences between countries [58]. About 9 % of the variance in self-rated health is only explained by the different countries (Interclass correlation − ICC _baseline model_ 0.091; Confidence interval (CI 95%) = (0.055, 0.147)). Thus, the central prerequisite for performing the multilevel analysis is fulfilled. Figure 2 illustrates the variance of self-rated health between countries.

The multilevel analysis was performed with R version 4.0.0 by the package lme4 [59]. For the analysis of mediation, the R package *mediation* was used, which allows one to examine mediations in multilevel models. The package is able to indicate average direct effects (ADE), average causal mediation effects (ACME), total (direct and indirect) effects, and the proportion of variables that pass through the mediator [60]. We tested the significance of indirect effects by using bootstrapping methods. Standardized indirect effects were calculated for each of 1.000 bootstrapping samples, and the 95% confidence interval was calculated by determining indirect effects with a confidence interval of 2.5% and 97.5% using the quasi-Bayesian method.

### 2.4. Specifications and Requirements

After extensive analysis and model comparisons, a varying intercept constant slope model was finally selected. Additional varying slopes of sunshine duration do not result in any model improvements. The model comparison can be seen in Table 1. AIC, BIC, and a Likelihood-Ratio-Test were used to determine whether the following model would be more suitable than the previous one.

Although it is sometimes recommended to center all independent variables in multilevel models on total mean [61], only the metric variables age and sunshine duration are centered on total mean in order to receive meaningful reference categories ($\bar{x}$_age_ = 46.475, $\bar{x}$_sunshine_ = 179.164). Furthermore, Mayerl and Urban advise against centering dummy variables on total mean from a statistical point of view [62]. In the present case, this means that the interpretation of the latitude line should be done exactly for the value dummy = 1 (outside interval by getting enough UVB exposure) and not over the entire statistical mean (which would be the case with centering). Because of our interest in the second level predictors, sunshine duration and latitude line, we decided against centering at group mean [63,64].

The number of variables on the macro-level matters in multilevel models. In total, 28 countries could be included in the analysis, whereby several regions and months were considered for each country and accordingly, 254 characteristics for sunshine hours are available. Table 2 lists how many data points representing sunshine hours were collected per country and from how many different regions they originate. It additionally shows the average skin color in the countries (measured by the region they were in) and the average domestic general government health care expenditures (measured by the survey years). It seems odd that Australia has a higher average skin color than South Africa. This is the case because the analyzed regions in Australia are closer to the equator than those in South Africa, with three out of five regions located directly on the south coast.

This means that our data are actually not structured by two, but four levels (country, region, survey date, individual). We decided to disregard the regional level because on the one hand regions do not have enough separate characteristics to make meaningful estimates and on the other hand variance of sunshine durations between regions within countries is very small. Integrating an additional third regional level would therefore only increase complexity and make interpretation more difficult. Survey time was recoded to seasons and accordingly included as a control variable at the individual level, but associated estimators play no particular role in this case.

In sum, values from 28 countries add to a total sum of 254 values of sunshine duration from 94 different regions, as different monthly data are available for sunshine duration per region, see Table 2. For correct estimation of standard errors in case of interest in contextual effects, at least 50 groups should be available for the macro-level [63]. This means that already 50 sunshine durations for the second level would be sufficient to assume correct standard errors.

A common problem with multilevel models is the multicollinearity between context variables [64]. In this model, multicollinearity plays no particular role when the variables skin color, average domestic general government health care expenditures, and season are removed. However, the problem of multicollinearity exists only if the standard errors are also distorted. Therefore, the variable average domestic general government health care expenditures was first added to the model without multicollinearity. Neither for this variable nor for the other two variables that were added and checked separately, could a bias be detected. 

There is one other requirement for mediation. It must be possible to explain the independent variable at least in part by the mediator. In our case, this means that the region in which one lives has an influence on the hours of sunshine. Theoretically, this is proven by the latitude line. There is another more controversial assumption with two schools of thought. One says that you need a statistically significant effect of the independent variable on the dependent variable, the other says that this is not essential [65,66]. As explained in hypothesis two, this should be the case from a theoretical point of view. During the mediation, the preconditions are checked statistically again.

Average marginal effects (AMEs) are estimated in order to make interpretation easier. AMEs allow us to not only observe the statistic significance of influence factors but also provide a measure for the strength of these effects. For more information, see [67]. Furthermore, this approach enables us to determine the strength of the possible mediator and compare it with the main effect. All interpretations are made without further mention of average and with the other variables kept constant, as well as considering counterfactual assumption of average marginal effect.

## 3. Results

### 3.1. Descriptive Statistics

Table 3 gives an overview of categorical variables and Table 4 shows the metric variables we used. At the individual level, about 28% of respondents stated that they did not consider their health to be good, whereas 72% said that they were at least well. In standardized surveys, there is often a perceived distortion in health status, which most likely occurs due to selection. Particularly sick people are not able to participate in surveys, which makes it seem as if the majority of people are doing well, which would explain the skewed distribution here. A total of 40% have high confidence in their health care system, while 60% have low confidence in it. Most people have never smoked (55%), while 21% are former smokers, about 13% smoke up to 10 cigarettes a day, and about 11% smoke more than ten cigarettes a day. A total of 38% of people engage in a great amount of sports, 33% rarely, and 29% never exercise. Additionally, 35% people have a low, 39% a medium, and 26% a high level of education. Finally, 55% of persons examined are female and 45% are male. A total of 94 locations (measured on the basis of latitudinal lines) are identified and 23 regions are between 35° N and 35° S parallels (31%) while 71 are outside of this range (69%). Australia, Chile, Japan, South Korea, and China belong to both latitudinal groups, separated at the 35th parallel. Apart from the regions where there are no seasons (with roughly 7%), the seasons are equally represented.

The standard deviation of 89 sunshine hours per month amounts to almost half (49%) of the average of 180 sunshine hours. This suggests that variance is sufficiently high. The health system differences measured by the Domestic general government health expenditure also show sufficient variance. In terms of skin color (added after region), it is apparent that most people tend to be white and fewer people are black, with the highest expression of the scale not occurring at all. We want to acknowledge that this sample of countries is probably not representative of all countries and especially for countries that are within 35° latitudes. The respondents are 47 years old on average, with a standard deviation of roughly 17 years. The hours worked appear to be quite low on average and have a large standard deviation. However, this is only the case because the persons who are unemployed are included in the variable.

### 3.2. Analysis without Control Variables But under Country Control

**H1.**  *The more sunshine in a region the better the health.*

A logistic multilevel model under control of countries shows a statistically significant influence of sunshine duration on self-rated health (AME = 0.019, *p* = 0.016, CI 95% = (0.004, 0.035), calculation is not shown separately in a table). There is a main effect between sunshine duration and self-rated health which corresponds to the assumed direction.

**H2.**  *Regions further away from the equator are associated with lower prospects for good population health.*

The latitudinal lines also have a statistically significant effect on self-rated health. People living above 35° N and below 35° S parallel are 1.5% less likely to be in the good health group, but not statistically significant (*p* = 0.317, CI 95% = (−0.044, 0.014), calculation is not shown separately in a table). This corresponds to the assumed direction. The second prerequisite for testing a mediation effect is thus fulfilled. The second controversial assumption for mediation is not present here, but we still look at the mediation in Hypothesis 3.

**H3.**  *Sunshine duration mediates the effect of latitude lines on population health.*

**Figure 3:**
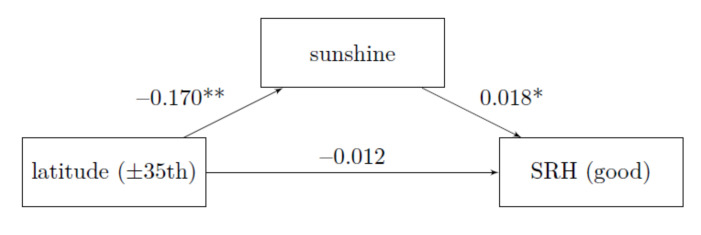
Mediation without control variables, ISSP 2011; average marginal effects; own calculation and representation; ** *p* < 0.01, * *p* < 0.05, proportion mediated: 14.14 %; *N* = 18,006.

The condition that the region in which one lives has a statistically significant effect on the hours of sunshine is given, see Figure 3 path latitude to sunshine. The effect of latitude lines on the likelihood of having good self-rated health was partially mediated via sunshine duration. As Figure 3 illustrates, the regression coefficient between latitude lines and self-rated health as well as the sunshine duration and the likelihood of good self-rated health was statistically significant.

The Average Causal Mediation Effect is calculated from −0.170 times 0.018 and is with −0.3 percentage points very small, but statistically significant (*p* = 0.012 CI 95% = (0.000, 0.012)). The proportion that goes through mediation is around 14.14%. The effect of latitudinal lines on self-rated health decreases from 1.5 to 1.2 percentage points.

### 3.3. Multivariate Analysis with Control Variables and Under Country Control

**Figure 4:**
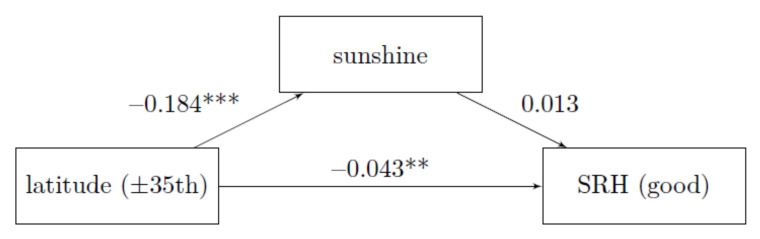
Mediation with control variables, Reference: ISSP 2011, average marginal effects, own calculation and representation, *** *p* < 0.001; ** *p* < 0.01, proportion mediated: 5.44 %; *N* = 18,006.

At this point, we first provide an overview of the full model (all control variables, but without mediation). That is, the influence of latitude and sunshine hours in relation to self-rated health while controlling for all other variables. This model is used for comparability. For the first hypothesis, a model is run without the influence of latitudinal lines, and for Hypothesis 2, a separate model is run without the influence of hours of sunshine to test for direct effects. In Hypothesis 3, mediation is tested with all variables and just as in the previous analysis in a separate Figure 4. Finally, all the results are related to each other in Section 4 Discussion.

**Figure 5:**
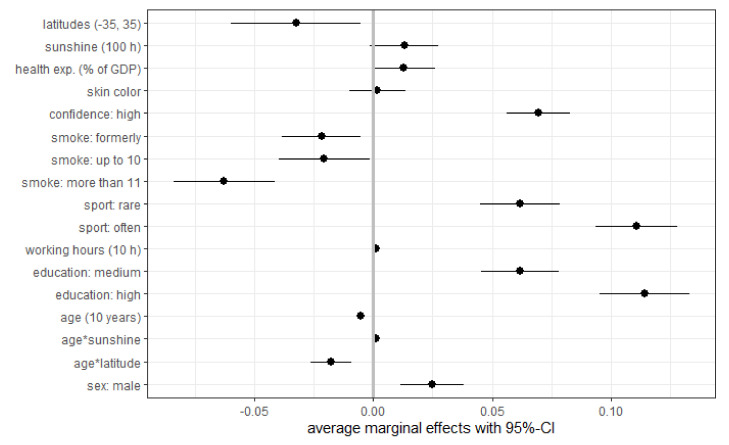
Coefficient plot of Varying intercepts constant slope model estimates including all control variables, but without mediation for comparison, health exp. (% of GDP) = domestic general gov. health expenditure (% of GDP), ISSP 2011, own calculation, *N* = 18,006.

In order to examine effects in more detail, the coefficients are shown in Table 5 and visualized in Figure 5. The R^2^ according to McKelvey and Zavoina can be interpreted the same way as the R^2^ from a (multiple) linear regression [68]. Regarding our model, this means that we can explain 18.67% of the variability in self-related health through independent variables. The interpretation of the following variables is carried out exclusively under the control of respective other variables included (ceteris paribus), which is not mentioned for every individual interpretation.

Table 5 now shows the full model without mediation. This means that the sunshine hours, as well as the latitude line, are in one model. The first two hypotheses are each tested without the other influencing variable, which is why the coefficients differ from the one in Table 5. This again shows relevant relationships between the variables.

The influences of the control variables are as expected: On the individual level, people with confidence in the health care system are 7 percentage points more likely to be in the good self-rated health group. People who smoke are up to 6 percentage points less likely to be in the good self-rated health group. If a person exercises regularly, the likelihood that they report being in an at least good health condition increases (up to eleven percent points). The higher the level of education, the higher the likelihood of reporting a good health status (up to eleven percent points) and men place themselves with 2.6 percentage points higher likelihood in the good self-rated health group. For every 10 h a person works, the probability of being healthy increases by 1.2 percentage points. The older a person is, the less likely they are to be among those who consider their health to be good to excellent (−4.2 percentage points for every ten years). However, these coefficients should not be underestimated since these are metric variables with a relative effect and not an absolute effect like the variables presented above. Age has the expected negative effect on health, but with sunlight, this effect can be mitigated. The older people are, the more they (might) benefit from being in a country with more sunshine hours. The interaction of age and latitude lines has the opposite effect. It amplifies the already negative effect of age. Both are statistically significant interaction effects (*p* = 0.000). Apart from smoking behavior (formerly: *p* = 0.011 and up to 10 a day: *p* = 0.027), all control variables are statistically significant at 0.1% level. None of the context-level control variables are statistically significant. Even if they do not distort the standard errors and estimators of the other variables when added, it does not seem reasonable to interpret them here, as they only serve to control the model.

**H1:** Sunshine duration shows a statistically significant test result: For every additional 100 h the probability to report having good health increases by 1.55 percentage points (*p* = 0.033, CI 95% = (0.001, 0.030), calculation is not shown separately in a table). This finding supports the previous analysis: By including control variables, the effect drops slightly from 1.90 percentage points to 1.55 percentage points and remains statistically significant. However, Table 5 (sunshine hours and latitude lines are included at once) shows that this effect is completely moderated by age.

**H2:** The latitude lines show a statistically significant test result at a 5% level: Persons living above 35° N and below 35° S parallel are 3.23 percentage points less likely to be in good health. This finding supports the previous analysis: By including control variables, the effect increases from 1.48 percentage points to 3.23 percentage points (more than twice) and is now statistically significant (*p* = 0.021, CI 95% = (−0.060, −0.005), calculation is not shown separately in a table), which was not the case before. Hypothesis 2 can be supported. The second prerequisite for testing a mediated effect is here also fulfilled.

**H3:** The condition that the place of residence has a statistically significant effect on the hours of sunshine is given, see Figure 4 path latitude to sunshine. Mediation is present in the analysis without control variables (see Figure 3) but the mediation disappears when all control variables are used (see Figure 4). The effect of Hypothesis 2 on the total effect remains unchanged.

The average causal mediation effect decreased from −0.3 to −0.2 percentage points and is no longer statistically significant (*p* = 0.084, CI 95% = (0.000, 0.084)). The proportion that goes through mediation is around 5.44%, previously it was 14%. This means that we chose the control variables sensitively, but there is no mediation left.

## 4. Discussion

This paper has examined the influence of sun exposure on self-rated health. Since this analysis required the variation of sunshine, an international comparative approach was chosen. We based our calculation on data from the ISSP 2011 which we complemented by adding international sunshine duration data from the German weather service. The intensity of sun exposure was operationalized by latitude lines and the duration of sunlight was processed on the level of the smallest possible regional differentiation. Hence, the differentiation of 94 regions in 28 countries was integrated into a multilevel analysis.

Theoretical considerations and previous empirical findings largely support our hypotheses, which suggest associations between duration of local sun exposure and distance from the equator as influence factors for health prospects. The bivariate regression to test Hypothesis 1 produces a statistically significant test result (AME = 0.016, *p* = 0.033), thus sunshine hours are correlated to subjective health. This suggests that a probability maximization of about 6 percentage points (1.55 percentage points times 4.12 sunshine hours = 6.39) is possible, which can be lifted if someone smokes more than eleven cigarettes a day or someone is ten years older. Therefore, this effect should not be overestimated. Similarly, under sole examination persons living above 35° N and below 35° S parallel (H2) show reduced self-rated health (AME = −0.032, *p* = 0.021). In an overall model (see Table 5), sunshine hours lose their effect, while latitude lines remain statistically significant. Sunshine duration mediates the effect of the latitude line on self-rated health only without control variables. When the control variables are included, there is no longer a mediation (H3). This is a very interesting result and shows how sensitive the control variables were chosen. Also, this indicates that location is a major determinant of the extent of sun exposure as well as a determinant on self-rated health.

Even when multivariate mediation is no longer present, the interaction effect between hours of sunlight and age is statistically significant: the older people are, the more they (might) benefit from being in a country with more sunshine hours. However, this may also be reverse causality, more healthy elderly can go out than sick elderly who are more likely to stay at home. Whereas we have controlled for physical fitness through control of sports practice and this should cushion the effect. It is also obvious that going out is more attractive when the sun is shining instead of raining. This result is an indication that the health-promoting effect for the elderly is present in connection with the hours of sunshine. However, the effect could also result from economic inequality leading to systematic differences in health and survival, or survivorship bias, which we unfortunately could not control.

In contrast, a region of residence near the equator has a much stronger influence on self-rated health. Since countries near the equator differ from more northern countries, the domestic general government health expenditure (% of GDP) was used as a macro-level control variable.

This study is not without limitations. It has to be noted that no precise information is available on how much time participants spent outdoors on each day during the survey period. This means that it was not possible to investigate how often a person was actually in the sun and had a chance to produce vitamin D. Factors that influence sun exposure, such as wearing UV-blocking clothing or using sunscreen, could not be considered either. Without these variables, it is difficult to prove possible vitamin D deficiency. An assumed lack of vitamin D was the main reasoning behind the second hypothesis and integrating more measures for vitamin D deficiency in future research could provide further insights. However, we have tried to capture this as best as possible using the given data by including hours worked as well as skin color in the analysis. Theoretically, it can also be assumed that the measurement errors addressed here should average out under otherwise identical conditions. Similarly, no information is available on possible vitamin D supplementation in the countries.

It should also be noted that the so-called Brandt Line separates the Global North from the Global South and states that people below 30° N, all other things being equal, have worse health. For America, Europe, and Africa, a comparable country classification would be obtained since we divide the countries at 35° latitude. For 35° South, it is recognized that there are few data points represented in the analysis that could compete with the rest. This is a possible alternative explanation of the effect of the region.

Furthermore, it was not possible to consider different health care systems in the countries as a substantively appropriate international classification of health care systems has not yet been developed [69]. It would certainly have been important to control for differences in the capacity of health care systems. To some extent, this circumstance could be brought into line with the respondents’ assessment of institutional trust in the local health care system, at least at the individual level. We also used domestic general government health spending (% of GDP) to best address this issue.

Systematic differences between the sample and the whole ISSP sample do not exist at first sight, but countries and also individual regions have been more or less arbitrarily excluded due to lack of data, which could possibly lead to distortion. The aforementioned multicollinearity is notwithstanding, the standard errors were not distorted and are within the acceptable range with regard to coefficient.

Due to the leeway given to respondents when asked about their health, their mood, which may be influenced by the weather, can also systematically affect their answer. Similarly, cultural differences between countries, for example, in terms of communicating with strangers or assessing one’s own health, may do so at the country level.

Answering the question of whether the influence of sunlight and health is due to vitamin D or to positive psychological effects is not easy because of the multitude of assumptions and confounding factors. It should be reemphasized how many proxies we had to choose to test our theoretical assumptions as best we could.

## 5. Conclusions

In summary, it can be noted that this study was able to show that sun exposure improves the variation on self-rated health across countries, even if this effect is quite small. The influence of the region plays a decisive role in contrast to the hours of sunshine. This result can be considered unsatisfactory as the distance to the equator cannot be influenced by health promotion measures (unless people migrate to other countries). However, older individuals seem to benefit from the hours of sunlight (subject to reverse causality), although there is no main effect of the sunshine hours. This suggests (subject to reverse causality) for older people that an increase in self-reported health might be achieved if they spend regular time in the sun.

## Figures and Tables

**Table 1:** Model Fit Comparison.

Model Selection	AIC ^1^	BIC ^2^	LR-Test ^3^
Baseline Model	21,357.928	21,365.726	
Varying Intercept Only	20,239.501	20,255.098	1120.43 **
Varying Intercept Constant Slope	18,253.620	18,432.985	2027.88 **
Varying Intercept Varying Slope	18,255.616	18,442.779	0.00

^1^ Aikaine-Information-Criterion, ^2^ Bayesian-Information-Criterion, ^3^ Likelihood-Ratio-Test (LR-Test) to previous model, ** *p* < 0.01, *N* = 18,006.

**Table 2:** Data points per country and country characteristics.

	Country	Region(s) per Country	Information on Regional Sunshine Hours per Month	Average Skin Color	Average Health Exp.	*N*
1	Australia	5	15	7	6.18%	712
2	China	3	11	3	2.32%	1146
3	Chile	5	9	3.80	3.19%	631
4	Croatia	1	2	3	6.39%	248
5	Czech Republic	4	8	1	5.87%	789
6	Denmark	4	8	1	8.57%	1105
7	Germany	3	18	1	8.18%	570
8	Finland	2	9	1	7.30%	422
9	France	2	10	1	7.87%	219
10	Great Britain	3	18	1	8.18%	499
11	Israel	1	6	4	4.41%	197
12	Italy	2	8	1	6.66%	182
13	Japan	8	8	2	8.89%	1049
14	Lithuania	3	6	1	4.58%	580
15	Netherlands	3	11	1	6.89%	155
16	Philippines	4	4	5	1.11%	1149
17	Poland	8	8	1	4.48%	543
18	Portugal	3	12	3	6.13%	397
19	Russia	6	6	1.92	3.00%	960
20	Sweden	1	3	1	8.80%	188
21	Switzerland	3	25	1	3.41%	664
22	Slovak Republic	1	3	1	5.41%	183
23	Slovenia	1	4	2	6.17%	225
24	Spain	4	11	2	6.60%	1291
25	South Africa	7	7	6.29	4.08%	2195
26	South Korea	3	9	2	3.52%	1030
27	Turkey	3	9	2	3.68%	507
28	Middle Atlantic (USA)	1	6	2	7.91%	170
**Sum**		**94**	**254**	**ø = 2.21**	**ø = 5.3%**	**18,006**

health exp. = domestic general government health care expenditures (as a % of gross domestic product), Source of skin color values: D. O’Neil (Behavioral Sciences Department, Palomar College, San Marcos, California), taken from Parra et al. [57].

**Table 3:** Description for categorial variables, *N* = 18,006.

Variable	Percentage	*N*
self-rated health		
- not good	28.01%	5043
- good	71.99%	12,963
confidence in health care system		
- low	60.42%	10,879
- high	39.58%	7127
smoking behavior		
- never	54.63%	9837
- formally	20.73%	3732
- up to 10 a day	13.20%	2376
- from 11 a day	11.45%	2061
sport behavior		
- never	28.99%	5220
- rare	33.03%	5948
- often	37.98%	6838
Education		
- low	34.59%	6229
- medium	39.70%	7149
- high	25.70%	4628
Sex		
- women	55.38%	9972
- men	44.62%	8034
latitude lines		
- 0–35th	30.93%	5570
- up to 35th	69.07%	12,436
Seasons		
- no season	6.78%	1220
- spring	22.63%	4074
- summer	21.73%	3913
- fall	22.96%	4134
- winter	25.91%	4665

**Table 4:** Description for metric variables.

Variable	*N*	Min	Max	$\bar{x}$	S
Sunshine hours	254	7	412	179.16	89.92
domestic general gov. health expenditure (% of GDP)	28	1.11	8.89	5.17	2.29
Skin color	94	1	7	2.77	2.03
Age	18,006	18	82	46.47	16.56
working hours	18,006	0	96	23.46	23.96

**Table 5:** Varying intercepts constant slope model estimates including all control variables without mediation for comparison.

Dependent Variable: Self-Rated Health (1 = Good)	AME	SE	CI 95%
latitudes (reference: −35th to 35th parallel)			
- ±35th	−0.032 *	0.014	(−0.060, −0.005)
sunshine (100 h)	0.014	0.007	(−0.000, 0.028)
domestic general gov. health expenditure (% of GDP)	0.013	0.007	(−0.001, 0.026)
skin color	0.002	0.006	(−0.010, 0.014)
confidence health care system (reference: no)			
- yes	0.074 ***	0.007	(0.061, 0.087)
smoke (reference: never)			
- formerly	−0.021 *	0.008	(−0.038, −0.005)
- up to 10 a day	−0.022 *	0.010	(−0.041, −0.002)
- more than 11 a day	−0.064 ***	0.011	(−0.085, −0.042)
Sport			
- rare	0.061 ***	0.009	(0.044, 0.078)
- often	0.110 ***	0.009	(0.093, 0.128)
working hours (10 h)	0.012 ***	0.002	(0.010, 0.015)
education (reference: low)			
- medium	0.059 ***	0.008	(0.043, 0.075)
- high	0.105 ***	0.010	(0.087, 0.124)
age (10 years)	−0.042 ***	0.004	(−0.049, −0.035)
sunshine*age	0.009 ***	0.002	(0.005, 0.014)
latitude*age	−0.018 ***	0.004	(−0.026, −0.009)
sex (reference: female)			
- male	0.026 ***	0.007	(0.012, 0.039)
Season (reference: no season)			
- spring	0.105 *	0.048	(0.010, 0.199)
- summer	0.106 *	0.048	(0.013, 0.199)
- fall	0.073	0.046	(−0.017, 0.164)
- winter	0.047	0.047	(−0.046, 0.140)
Log-Likelihood*_baseline model_*		−10,117.800	
Log-Likelihood*_full model_*		−9103.826	
R^2^*_McKelvey_*_&*Zavoina*_		18.90%	

Note: *** *p* < 0.001; * *p* < 0.05, N_Level1_ = 18,006; N_Level2_ = 28, with 254 sunshine durations and 94 regions (latitude lines).

## Data Availability

Data: The ISSP data can be accessed on the following homepage: http://www.issp.org/data-download. The data of the German Weather Service can be viewed here: https://opendata.dwd.de/climate_environment/CDC/observations_global/CLIMAT/monthly/qc/sunshine_duration/historical/. We are happy to provide our data extension to the ISSP 2011 individually. Please contact the corresponding author for more information.
